# Application of light in delirium: a bibliometric analysis

**DOI:** 10.3389/fneur.2025.1549396

**Published:** 2025-07-02

**Authors:** Yan Zheng, Yongke Zeng, Xiaoqin Luo, Liangxiang Xia, Jing Chen, Sujuan Lei, Silin Zheng

**Affiliations:** ^1^School of Nursing, Southwest Medical University, Luzhou, Sichuan Province, China; ^2^Department of Hepatobiliary Surgery, Affiliated Hospital of Southwest Medical University, Luzhou, Sichuan Province, China; ^3^Department of Nursing, Affiliated Hospital of Southwest Medical University, Luzhou, Sichuan Province, China

**Keywords:** delirium, light, bibliometric analysis, Citespace, VOSviewer, R-bibliometrix

## Abstract

**Background:**

Delirium is an acute neurocognitive disorder prevalent among older adults and critically ill patients. Light therapy has garnered growing interest as a potential non-pharmacologic intervention for delirium management. This study conducted a bibliometric analysis of Web of Science Core Collection publications to systematically evaluate research progress and evolutionary trends in light therapy for delirium.

**Materials and methods:**

Publications were retrieved from the Web of Science Core Collection. Bibliometric parameters including temporal trends, geographical distribution, institutional affiliations, journal impact, author productivity, keyword co-occurrence, and thematic clusters were analyzed using VOSviewer, R-bibliometrix package, and CiteSpace.

**Results:**

Bibliometric analysis demonstrated a steady increase in annual publications, with the United States (US) contributing the highest output. Johns Hopkins University emerged as the most productive institution, while Yahya Shehabi and Melissa P. Knauert were identified as leading contributors. *Nursing in Critical Care* had the highest number of publications. Key research domains included” sleep,” “intensive-care-unit,” “mechanically ventilated patients,” “critically-ill patients,” “light,” and so on.

**Conclusion:**

The application of light in delirium is an evolving field, and further research is needed to build a well-established structural system to provide a scientific basis for clinical application. Future research should focus on specific populations, multifactorial interventions, and basic science research to strengthen the application of light therapy in the treatment of delirium.

## 1 Introduction

Delirium is a neuropsychiatric syndrome characterized by disrupted perception, cognition, psychomotor activity, and circadian rhythms, which poses significant clinical risks (1, 2). It is most common in the elderly population, especially in patients with severe physical illnesses, and is also a common complication in critically ill and postoperative patients, affecting 70–87% of Intensive Care Unit (ICU) patients (3, 4) and 10–50% of postoperative patients (5). Delirium not only impairs the patient’s physical and mental health but also leads to prolonged hospitalization, increased consumption of healthcare resources, and an additional burden on families and healthcare professionals. Therefore, timely prevention and treatment of delirium is particularly important. Currently, the treatment of delirium is mainly divided into two categories: pharmacological treatment and multimodal nonpharmacological treatment. Professional guidelines and consensus recommend the use of multicomponent nonpharmacological treatment measures for the prevention or treatment of delirium (6, 7). Combining oxygen therapy (8), sleep regulation, and ICU room design (9) has been effective in intervening for delirium. Thus, light therapy is gaining attention as a potential nonpharmacologic multicomponent intervention.

Research indicates that light therapy might affect the onset and outcome of delirium by modulating circadian rhythms and melatonin levels. Thus, the number of studies on its application as an intervention for delirium management is increasing. With the increasing aging of the global population, technological innovations, enhanced interdisciplinary communication, and the global rise in critical illnesses (10, 11), the research and application of light-based therapies in preventing and ameliorating delirium are advancing and deepening. Therefore, it is particularly important to conduct a systematic review and analysis of existing studies.

In this study, we intend to analyze the research application of phototherapy in the field of delirium by using bibliometrics to reveal the important authors, journals, and countries in the field, and to provide readers with a more intuitive understanding of the current status of the field’s application and development trend as a reference basis.

## 2 Methodology and data source

### 2.1 Bibliometric analysis

Bibliometric analysis is a research approach that systematically analyzes the development trend of a discipline relying on quantitative data. It constructs a multidimensional knowledge network to uncover the research characteristics of the field by extracting metadata from academic databases, encompassing information about authors, institutions, countries, keywords, journals, and references (12, 13). This study employs four core analysis modules of the method: (1) chronological analysis: monitoring changes in the number of publications to quantify the developmental trajectory of the field; (2) collaborative network analysis: analyzing the intensity of collaboration among countries, institutions, and authors to identify core research groups; (3) thematic clustering analysis: identifying research hotspots and frontier directions through the detection of keyword co-occurrences and sudden emergences; (4) Impact assessment: integrating citation frequency and journal categorization to evaluate the academic contribution of significant findings. Together, these analytic dimensions serve the core objective of this study - to reveal the research progress, collaboration patterns, and future trends of light therapy in the field of delirium intervention.

To achieve these analyses, three types of bibliometric tools were integrated into this study: VOSviewer (version 1.6.20) for constructing national/institutional collaborative networks and keyword co-occurrence mapping. CiteSpace (version 6.3. R1) for detecting research hotspot shifts through burst word detection and time-series evolution analysis; R-bibliometrix package (version 4.0.0) for data cleansing, basic statistical analysis, and visualization verification.

### 2.2 Data

**Figure 1:**
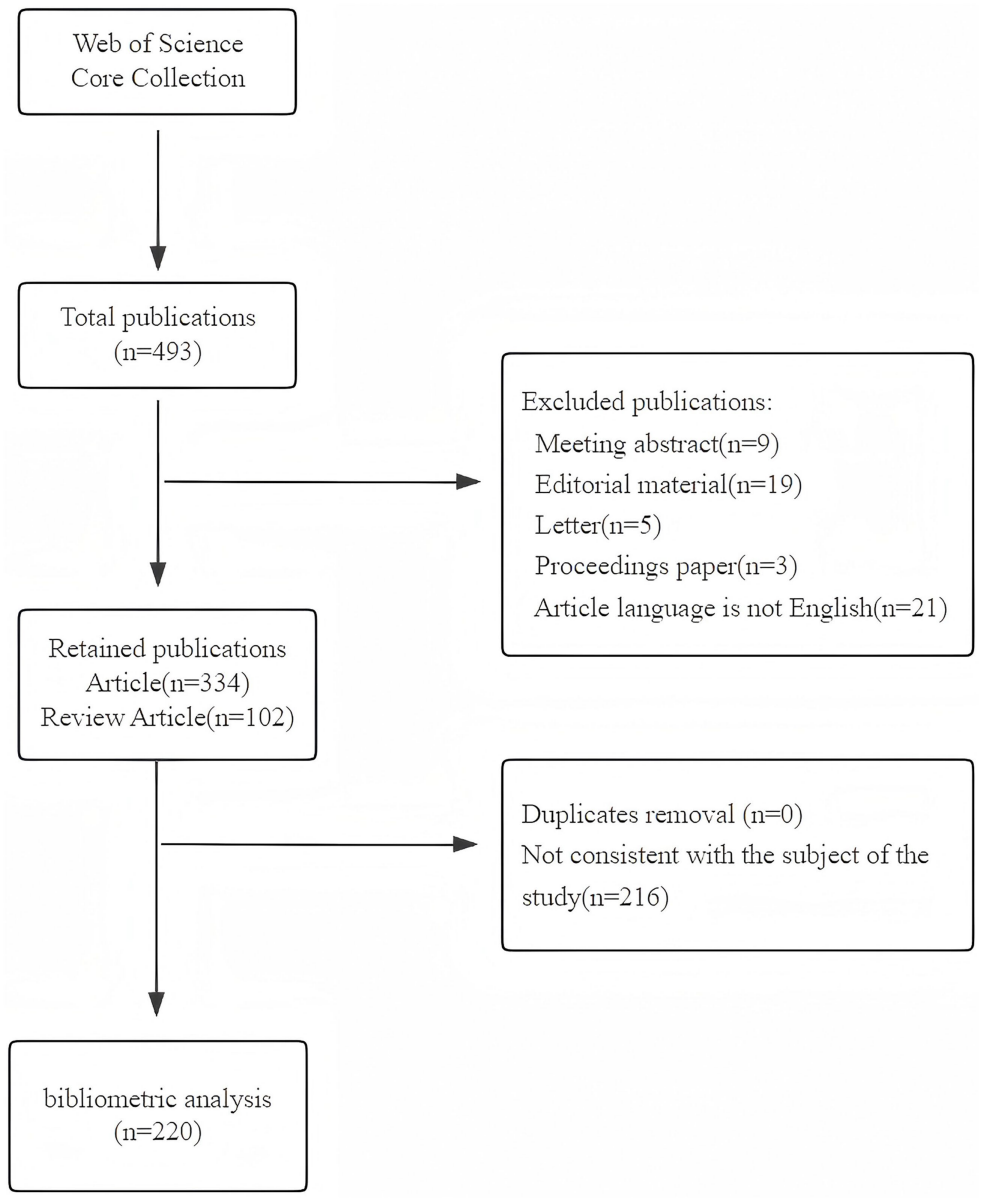
Flowchart for the publication selection included in this study.

We selected the Web of Science core collection as the data source and chose the Science Citation Index Expanded (SCI-EXPANDED) and Social Sciences Citation Index (SSCI) as the indexes to guarantee the comprehensiveness and accuracy of the data. A literature search was conducted on a single day (June 19, 2024) because of the rapid updating of the databases. The publication period of this study was set from 1900 to 2024. Considering that “light” and “light therapy” are similar in form, the final search strategy was Topic (TS) = (delirium) AND TS = (Light OR “light therapy”). After the subject search, a total of 493 articles were retrieved. Initially, 436 articles were selected based on the literature type for English articles and reviews. Finally, 220 results were obtained as some literature reports were not relevant to the research on the application of light in delirium and were manually excluded. Figure 1 shows the search process for the bibliometric analysis.

## 3 Results

### 3.1 Descriptive statistics

The 220 papers used in this study came from 1,370 authors from 563 institutions in 141 countries, published in 129 journals, and cited 7,508 citations from 2,297 journals.

### 3.2 Quantities and trends of publications output

**Figure 2:**
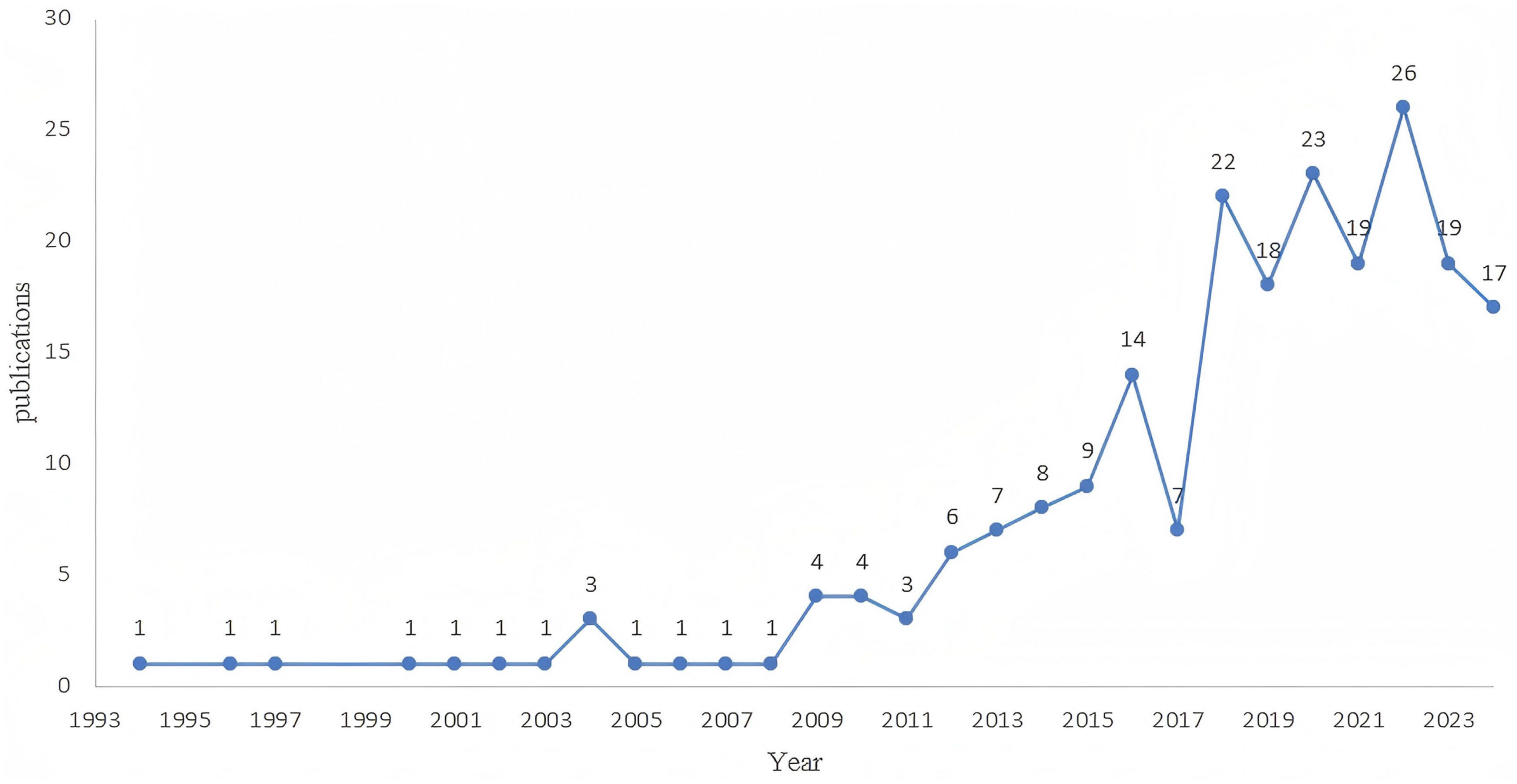
Quantities and trend of publications output.

Figure 2 illustrates the publication and growth dynamics over time of the research literature related to the application of light to the field of delirium. The earliest article was published in 1994, and subsequently, the number of relevant articles exhibited a steady increase. Based on trends in the number of publications and by considering key advancements in the field, we have identified three distinct growth phases. The first phase spanned from 1994 to 2008, during which the number of articles published annually remained at 1. There was 1 small peak during this period, i.e., the number of articles published reached 3 in 2004, which was in the budding stage. The second stage extended from 2009 to 2016. During this stage, the number of articles published slowly increased to reach 14 articles in 2016, reflecting the timeline of the development stage. The third stage ranges from 2017 to 2024. In this stage, the output of articles has significantly increased, and the number of articles has stabilized at approximately 20 per year. This indicates that an increasing number of scholars are paying attention to this field, highlighting the growing significance of light in delirium research and demonstrating a flourishing trend.

### 3.3 Analysis of authors

**Figure 3:**
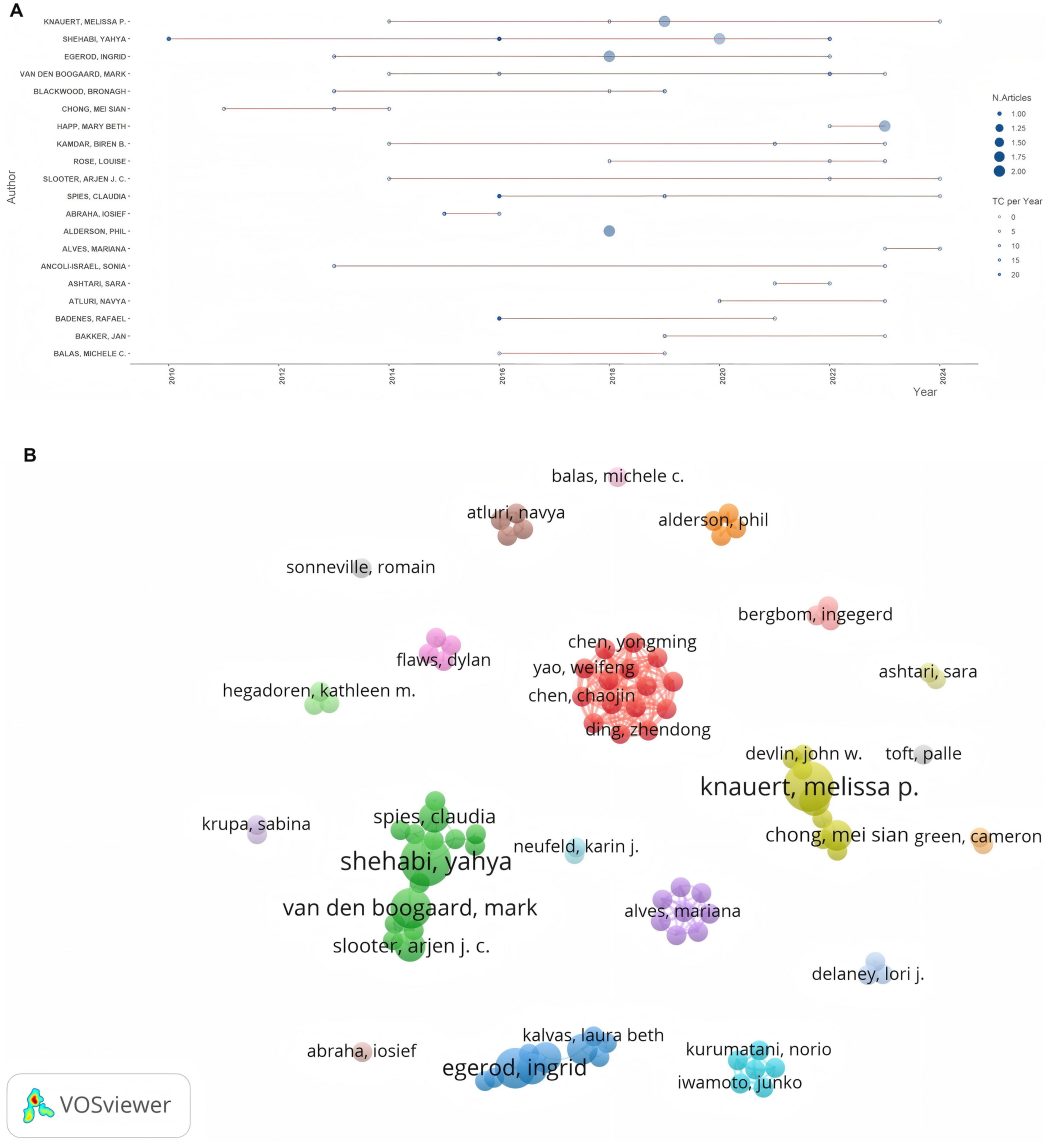
**(A)** Authors’ production in over time. **(B)** Author cooperation network.

In accordance with Price’s law, authors who have published two or more articles are regarded as core authors in the field, resulting in a total of 96 core authors. Table 1 presents authors who have published more than three papers on the application of light in delirium-related fields. Among them, Shehabi, Yahya ranked first with the greatest number of studies (*n* = 5) and the highest H-index (4), demonstrating his significant influence in the research field. Knauert, Melissa P. also published five articles, tying for first place with Shehabi, Yahya; subsequently, Van den Boogaard, Mark (*n* = 4) and Egerod, Ingrid (*n* = 4) followed. As evidenced by the “Author Production over Time” graph (Figure 3A) generated by Bibliometrix software, Shehabi, Yahya, and Knauert, Melissa p have been actively engaged in this area for over 10 years, and their research endeavors persist. In addition, the co-author network was visualized using VOSviewer (Figure 3B), and a total of 20 clusters were identified based on the proximity of co-author collaborations. Shehabi, Yahya and Knauert, Melissa P. represent the largest nodes, and Van den Boogaard, Mark, and John W Devlin, who collaborate closely with them, also possess a high academic reputation in this field. This indicates that collaboration among leading scholars has facilitated the development of the field of light applied to delirium research. Notably, the scarcity of connections between different clusters, implies that scholars in this field predominantly engage in collaboration within academic institutions or national boundaries, exhibiting relatively limited cross - institutional and international collaboration.

**Table 1:** The top authors with the most research articles.

No	Author	Affiliation	Publications	Citations	h-index
1	Shehabi, Yahya	Universiti Malaya	5	587	4
2	Knauert, Melissa P	Yale University	5	43	3
3	Van den Boogaard, Mark	Radboud University Nijmegen Medical Center	4	120	3
4	Egerod, Ingrid	University of Copenhagen	4	120	4
5	Happ, Mary Beth	Ohio State University	3	13	3
6	Spies, Claudia	Charité Universitätsmedizin Berlin	3	263	3
7	Blackwood, Bronagh	Queens University Belfast	3	135	3
8	Chong, Mei Sian	Tan Tock Seng Hospital	3	68	3
9	Rose, Louise	University of Toronto	3	27	3
10	Slooter, Arjen J. C.	Utrecht University	3	20	3
11	Kamdar, Biren B.	University of California, San Diego	3	36	3

### 3.4 Analysis of journals

**Figure 4:**
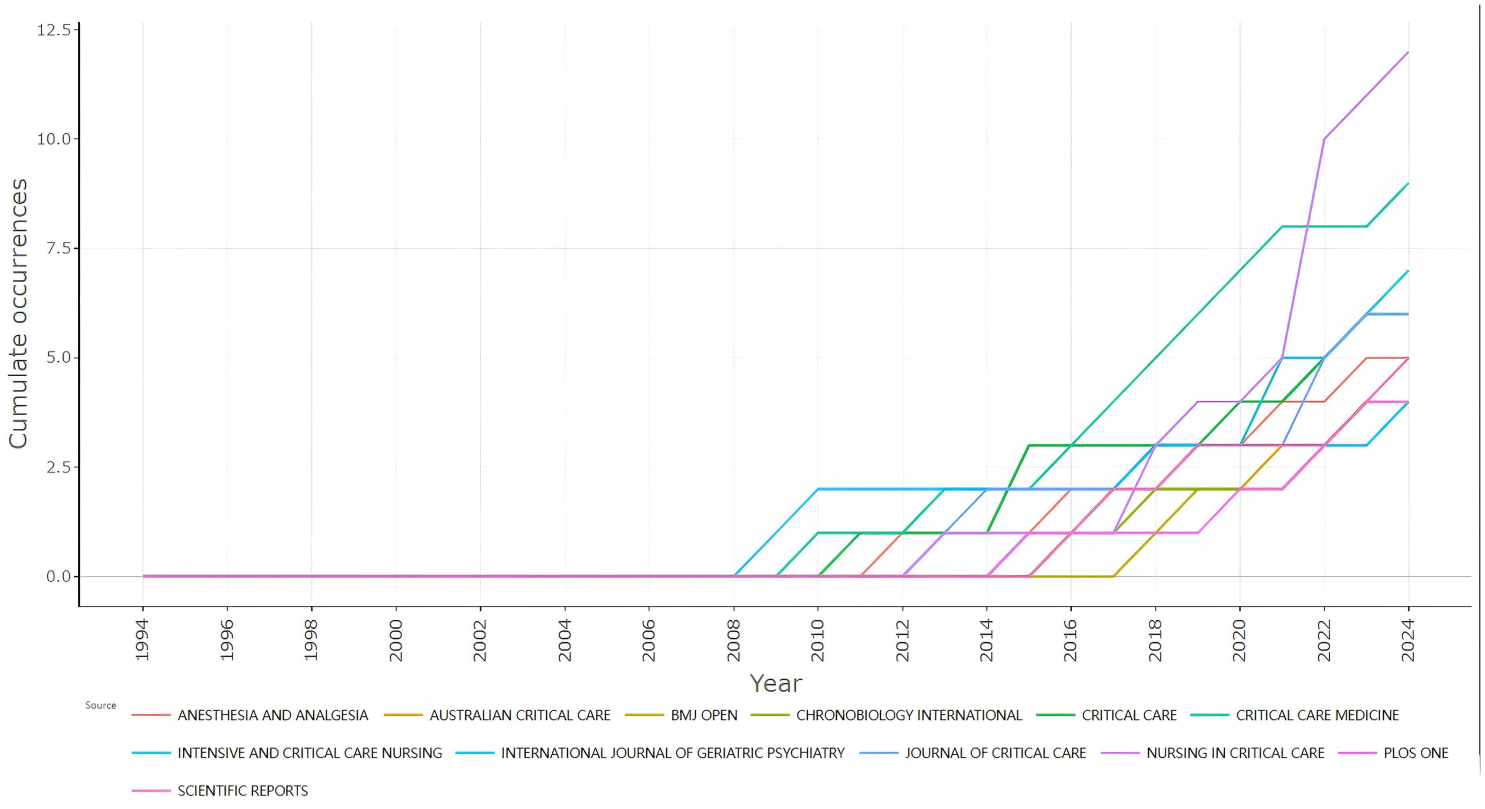
Journals’ production in over time.

Upon analyzing the journal publications pertaining to the application of light in the field of delirium treatment, a total of 129 journals featuring research articles in this domain were identified. Notably, the majority of these journals centered on the medical field, encompassing critical care medicine, anesthesiology, and nursing. Table 2 presents the top 10 journals with the largest number of published articles, which together published about 28.18% of the articles in this field. Among them, *Nursing in Critical Care* published the greatest number of articles (*n* = 12), accumulating 149 citations, yet ranked seventh in the Impact Factor (IF) ranking. The second- and third-ranked journals in terms of publication volume were *Critical Care Medicine* (*n* = 9) and *Intensive and Critical Care Nursing* (*n* = 7), respectively. *Critical Care Medicine* emerged as the most-cited journal, amassing 555 citations, with an average of 61.67 citations per article. The top three journals were all categorized in the first quartile (Q1) of the Journal Citation Reports (JCR). In this ranking system, Q1 denotes the top 25% of journals by impact factor within their respective subject categories, signifying high academic influence. As illustrated in Figure 4, Journal output over time, six of the top 10 journals in terms of article quantity initiated a gradual shift toward focusing on research related to the application of light in delirium treatment post-2015, and the number of research articles published in these journals displayed an overall upward trend.

**Table 2:** The top 10 journals with the most articles.

No	Journal	Publications	Citations	Average citation/publication	IF (JCR2023)	JCR quatile
1	Nursing in critical care	12	149	12.42	3.0	Q1
2	Critical care medicine	9	555	61.67	7.7	Q1
3	Intensive and critical care nursing	7	134	19.14	4.9	Q1
4	Critical care	6	252	42	8.8	Q1
5	Journal of critical care	6	69	11.5	3.2	Q2
6	Anesthesia and analgesia	5	179	35.8	4.6	Q1
7	Scientific reports	5	84	16.8	3.8	Q1
8	Australian critical care	4	76	19	2.6	Q2
9	Bmj open	4	27	6.75	2.4	Q1
10	Chronobiology international	4	132	33	2.2	Q2

### 3.5 Analysis of the countries

**Figure 5:**
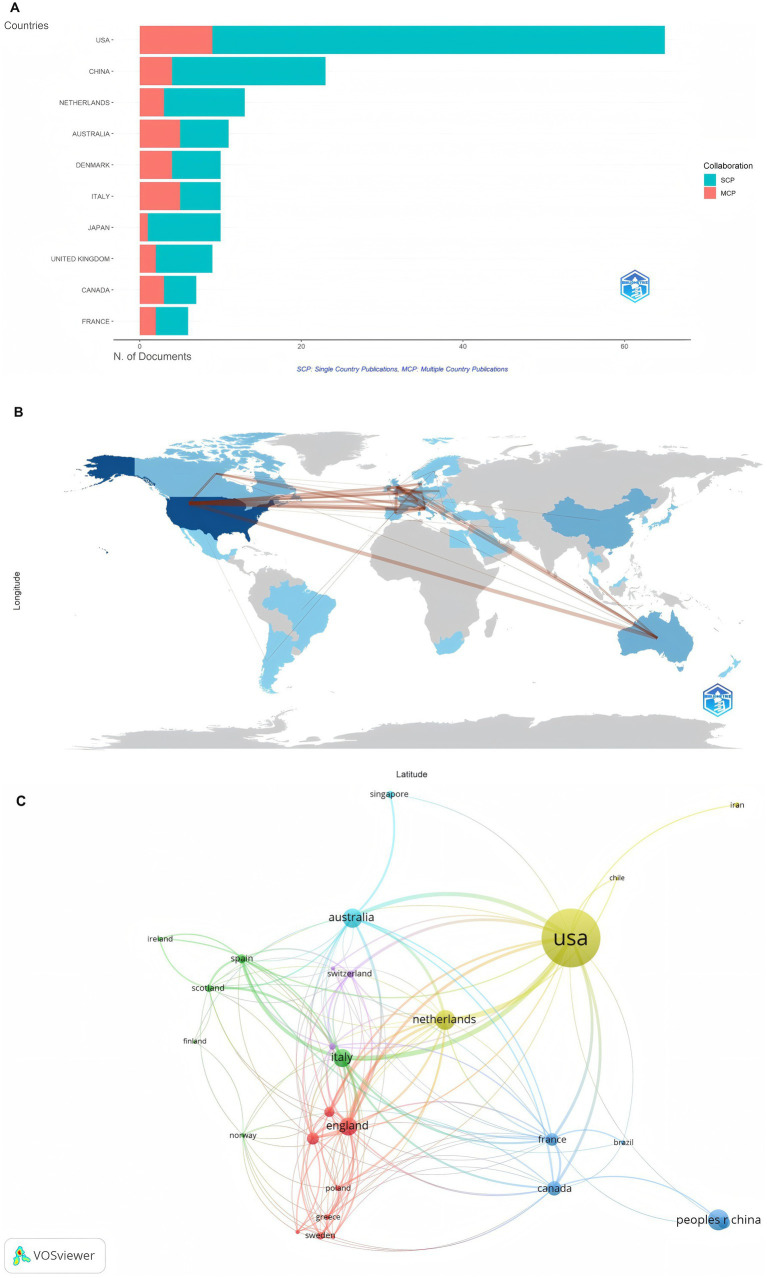
**(A)** The top 10 countries in output (corresponding authors included). **(B)** Countries’ collaboration world map. **(C)** Analysis of collaborative network of countries.

A total of 141 countries contributed publications, and Table 3 lists the top 10 countries in terms of the number of publications. The United States (US) ranked first in terms of the number of author publications, with 82 publications, followed by the United Kingdom (UK; *n* = 24) and China (*n* = 23). In terms of citations, the US led the ranking with 2,666 citations, followed by the UK with 1,000 citations and Italy with 722 citations, respectively. France had the highest average number of citations, approximately 47, whereas China, despite ranking among the top three in terms of the number of publications, had fewer citations and a lower average citation count. Figure 5A illustrates the national article output categorized by the corresponding author’s country, which comprises Multiple Country Publications (MCP) and Single Country Publications (SCP). MCP refers to articles co-signed by authors from multiple countries, reflecting the current situation of international cooperation and academic exchange; SCP, on the other hand, refers to the number of articles in which all authors are from the same country. As evidenced by the figure, most of the countries’ research is conducted in their own countries. However, Figures 5B,C reveal that there is still a certain degree of international cooperation and academic exchange between countries. MCPs constitute approximately one-third of the total in Figure 5A, thereby indicating the presence of collaborative exchanges. In Figure 5B, denser connections are observed between North America, Europe, and Oceania. In Figure 5C, each circular node represents a country; the size of the node represents the contribution of the country’s publications, and the links between the nodes signify the collaborative relationships between the countries, with thicker links signifying more and deeper cooperation between the countries. The US has the greatest total link strength in this research field and maintains relatively close collaborative relationships with China, the UK, the Netherlands, Australia, Canada, and Italy. Among these countries, the UK, Italy, and Australia lead in the number of citations per article, indicating that the research of all these countries has a certain degree of influence in the field. Overall, the contribution of articles varies among countries, and enhanced international cooperation and exchange are imperative for elevating the quality and impact of research.

**Table 3:** Top 10 country in terms of the number of publications.

No	Country	Publications	Citations	Average citation/publication	Total link strength
1	United States	82	2,666	35.51	34
2	United kingdom	24	1,000	41.67	25
3	China	23	281	12.22	4
4	Netherlands	21	613	29.19	22
5	Australia	20	764	38.2	20
6	Italy	18	722	40.11	23
7	Canada	14	424	30.29	18
8	France	12	567	47.25	18
9	Denmark	11	475	43.18	14
10	japan	11	331	30.09	2

### 3.6 Analysis of affiliations

**Figure 6:**
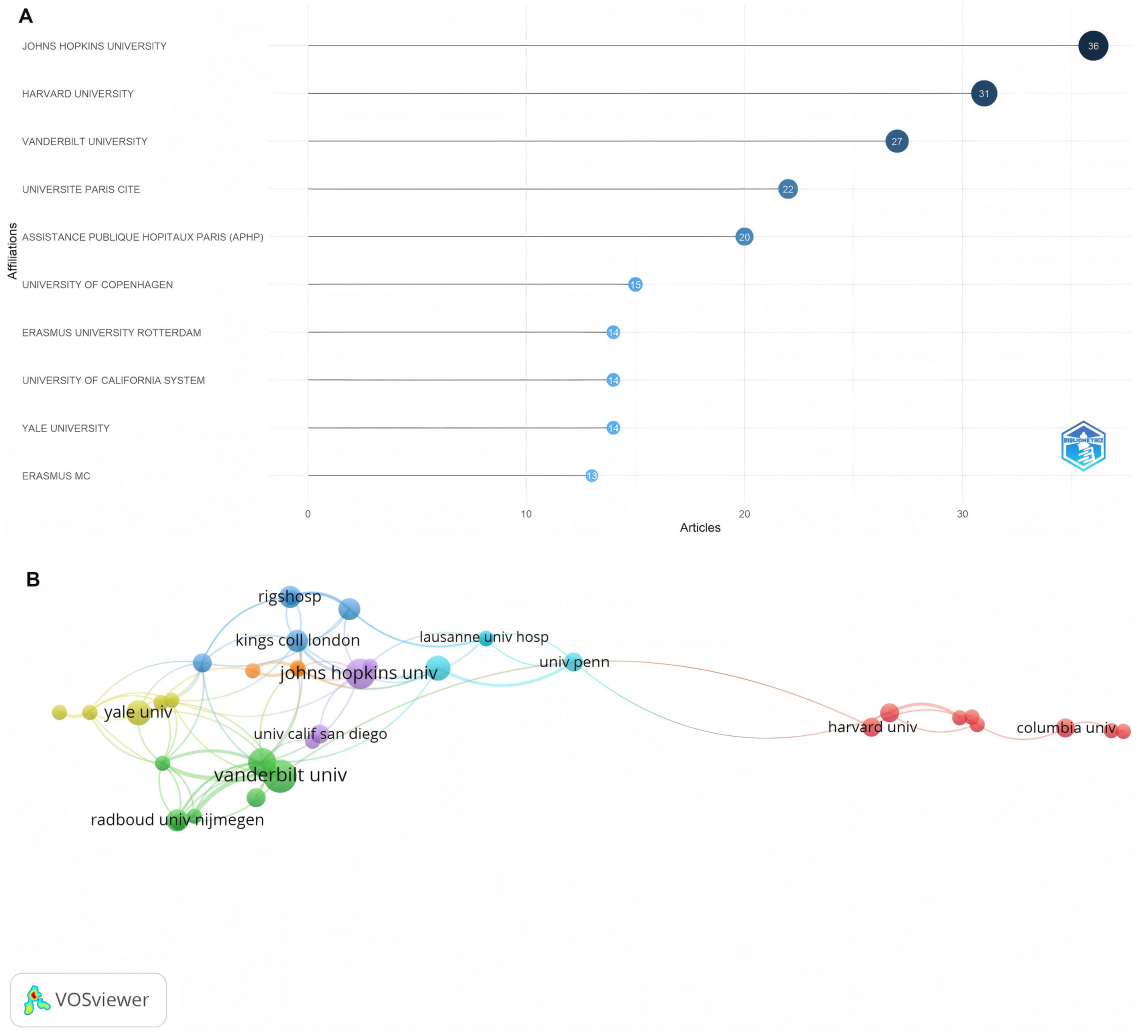
**(A)** The top 10 affiliations with the most publications. (B) Cooperation networks across affiliations.

A total of 563 institutions participated in the research field pertaining to the application of light to delirium, and the majority of these institutions were universities. Figure 6A depicts the top 10 institutions boasting the highest number of scholarly outputs. Among these institutions, Johns Hopkins University ranked first, having published 36 papers; Harvard University came in second with 31 papers, and Vanderbilt University secured the third position with 27 papers. Notably, approximately one-third of the participating institutions released only one paper. The institutional collaboration network diagram generated by VOSviewer (Figure 6B) reveals that inter-institutional collaborations are predominantly concentrated in a select few universities, including Johns Hopkins, Vanderbilt, Monash, and Yale. However, these institutions exhibit limited close collaboration with one another. Although Harvard ranks among the top three in academic output, it engages in minimal collaboration with other institutions, forming a more independent red cluster characterized by a distinct regional nature.

### 3.7 Analysis of citations

Through co-citation analysis (i.e., the frequency with which two documents are simultaneously cited by subsequent studies), we can obtain a more profound understanding of the pivotal papers that are highly cited within a specific research field. Table 4 presents the top 10 most-cited articles, the majority of which are from journals in the field of critical care medicine. Among them, a research article titled *“Delirium as a Predictor of Mortality in Mechanically Ventilated Patients in the Intensive Care Unit”* by Ely E. Wesley et al. (14) has garnered significant attention due to its findings in prospective cohort studies - delirium is an independent predictor of 6-month mortality risk and prolonged hospital stay in mechanically ventilated patients. This article has furnished valuable information for subsequent delirium research by scholars, with 43 citations. This was followed by studies by Barr Juliana et al. (with 31 citations) and John W Devlin et al. (with 29 citations), both of which offer clinical practice guidelines regarding delirium prevention and management.

**Table 4:** The top 10 cited references.

No	Author	Article title	Journal	Cited	Year
1	Ely E. Wesley	Delirium as a Predictor of Mortality in Mechanically Ventilated Patients in the Intensive Care Unit	JAMA	43	2004
2	Barr Juliana, et al.	Clinical Practice Guidelines for the Management of Pain, Agitation, and Delirium in Adult Patients in the Intensive Care Unit	Critical Care Medicine	31	2013
3	John W Devlin, et al.	Clinical Practice Guidelines for the Prevention and Management of Pain, Agitation/Sedation, Delirium, Immobility, and Sleep Disruption in Adult Patients in the ICU	Critical Care Medicine	29	2018
4	Ely E. Wesley,et al.	Delirium in Mechanically Ventilated Patients	JAMA	28	2001
5	Elliott Rosalind, et al.	Characterisation of sleep in intensive care using 24-h polysomnography: an observational study	Critical care	27	2013
6	Pandharipande P. P, et al.	Long-Term Cognitive Impairment after Critical Illness	The New England Journal of Medicine	27	2013
7	FREEDMAN NEIL S, et al.	Abnormal Sleep/Wake Cycles and the Effect of Environmental Noise on Sleep Disruption in the Intensive Care Unit	American Journal of Respiratory and Critical Care Medicine	26	2001
8	Taguchi Toyoe, et al.	Influence of bright light therapy on postoperative patients: A pilot study	Intensive and Critical Care Nursing	26	2007
9	Pisani Margaret A, et al.	Sleep in the Intensive Care Unit	American Journal of Respiratory and Critical Care Medicine	25	2015
10	Patel J, et al.	The effect of a multicomponent multidisciplinary bundle of interventions on sleep and delirium in medical and surgical intensive care patients	Anesthesia	24	2014

**Figure 7:**
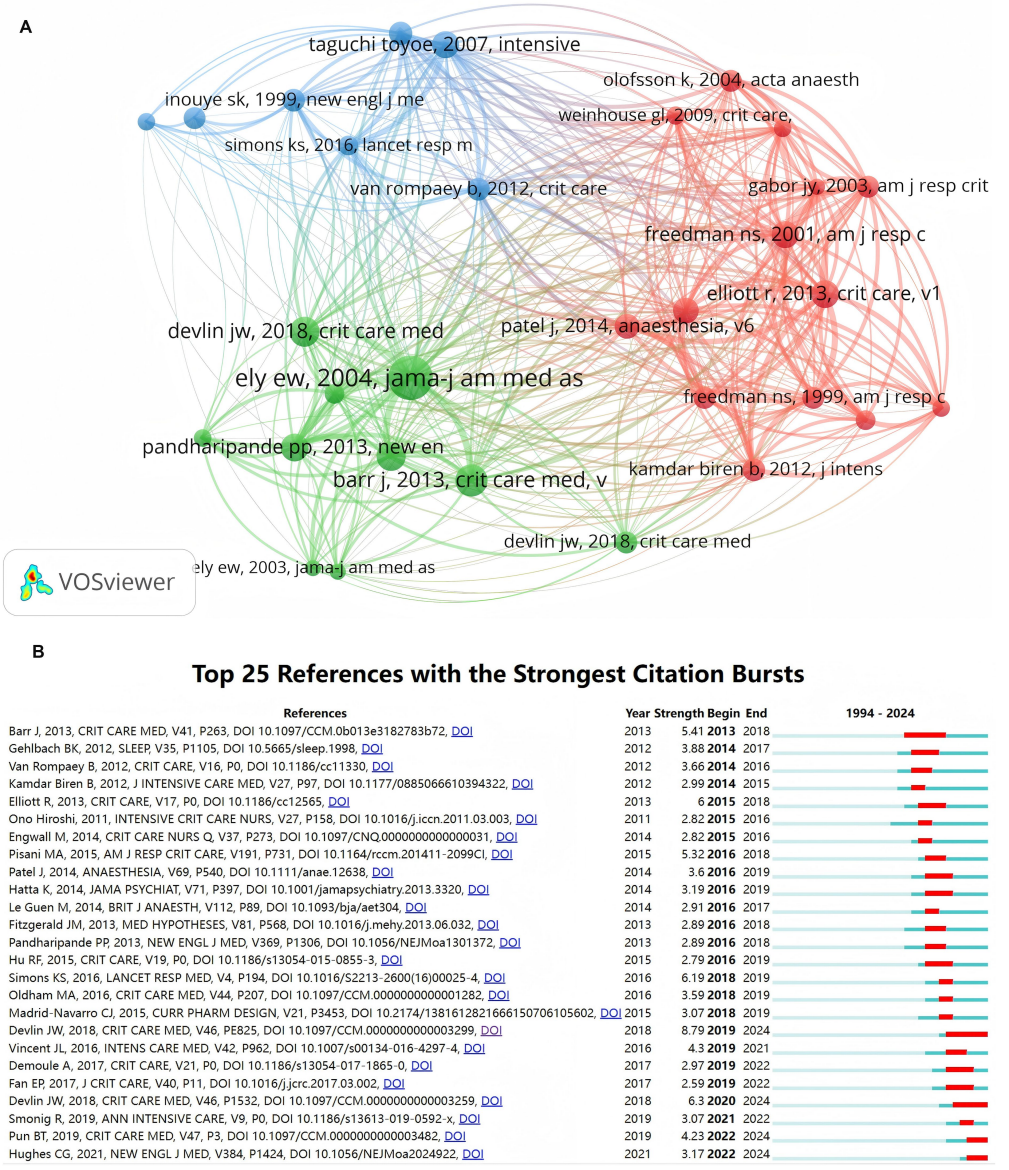
**(A)** The network of co-cited references. **(B)** Top 25 references with strongest citation bursts. Parameters for calculating burst intensity: *γ* = 0.5, minimum burst length = 2 years; red line segments indicate the active citation period of the literature.

Figure 7A illustrates the co-citation network graph of references generated by VOSviewer software. In this graph, the size of the nodes reflects the citation frequency of the literature, the color clustering signifies the differences in research topics, and the thickness of the connecting lines indicates the co-citation intensity. The top 25 documents with the highest citation burst rate were further demonstrated by CiteSpace’s burst detection algorithm (with parameters: *γ* = 0.5, minimum burst length = 2 years; Figure 7B).

The first citation burst occurred in 2013 for the article *“Clinical Practice Guideline for the Management of Pain, Agitation, and Delirium in Adult Patients in Intensive Care Units”* (15) authored by Barriana Juliana et al. It is worthy of note that the article by John W Devlin et al. is of particular significance in terms of citation burst intensity(a metric for measuring the short-term impact of the literature), with an intensity value of 8.79, and there are two citation bursts, the other of which is the executive summary of the same research team. As depicted Figure 7B, the citation burst cycle of the two articles by John W Devlin et al. endured until 2024, which aligns with the third stage of the literature publication timeline, suggesting that their study is highly congruent with the current research trend. As of 2024, there have been four citation bursts, signifying that research on the application of light to delirium is ongoing.

### 3.8 Analysis of keywords

Keywords serve as a distillation of the core content within an article and they can reveal research hotspots and trends in scientific fields through co-occurrence analysis. We carried out a co-occurrence analysis of keywords by utilizing the VOSviewer tool aiming to gain a more comprehensive understanding of these implications and trends.

**Figure 8:**
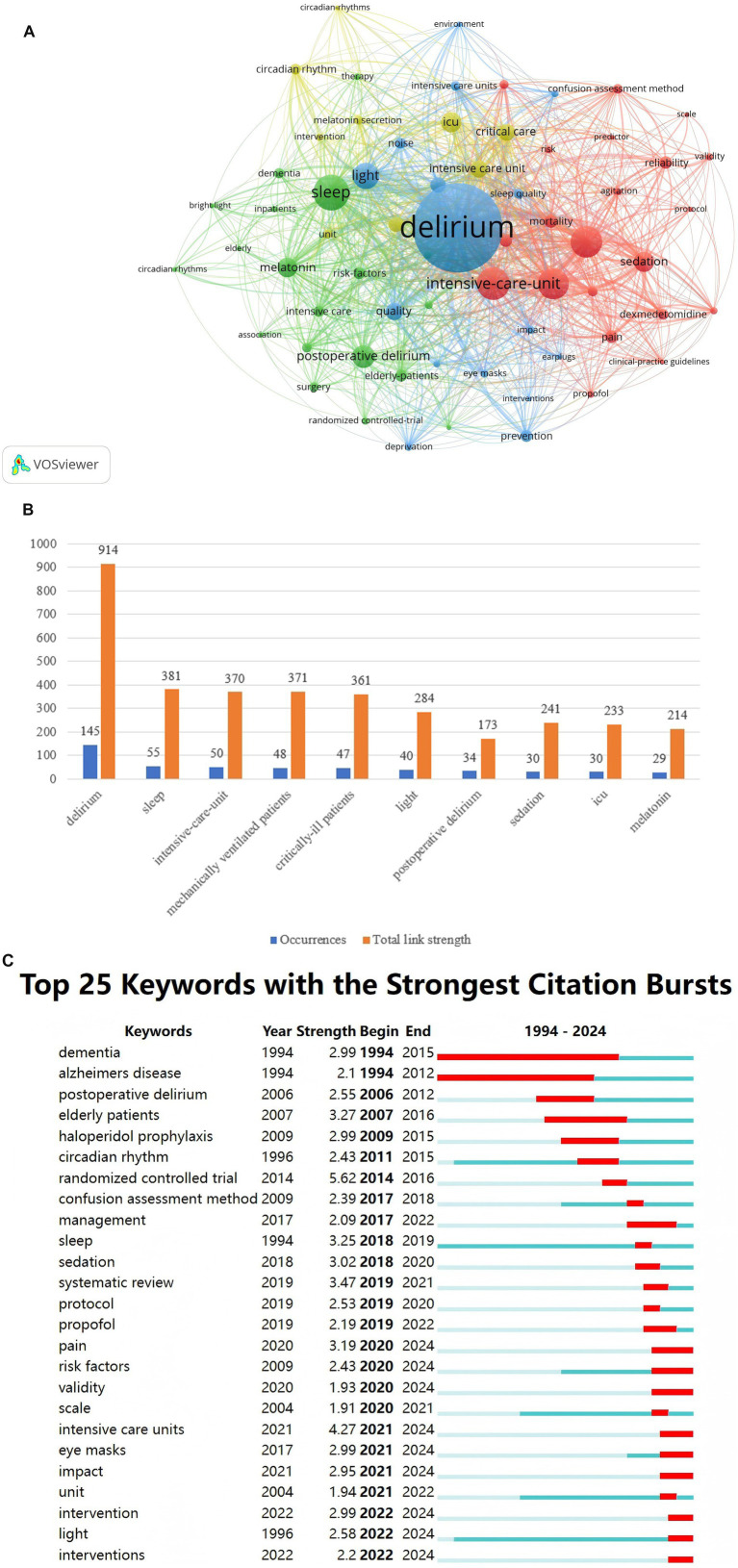
**(A)** Keyword structure co-occurrence network. **(B)** Top 10 keywords. **(C)** Top 25 keywords with strongest citation bursts.

In co-occurrence analysis, the size of the nodes indicates the frequency of keyword occurrence, the thickness of the connecting lines reflects the co-occurrence intensity (i.e., the total number of times two keywords co-occur within the same literature; the thicker the connecting line, the greater the frequency of co-occurrence between the two keywords), and the color clustering signifies research topic relevance. Through the network visualization of keyword co-occurrences (as depicted in Figure 8A), we discovered that the keyword “delirium” had the highest frequency of occurrence (145) and the greatest total link strength (914; see Figure 8B). Total link strength represents a measure of the cumulative strength of the connections between a particular keyword and all other co-occurring keywords. The greater the total link strength, the more central the keyword is within the network, and the stronger its relationship is with other keywords, which is in line with the centrality of “delirium” as a core theme and its central position in the co-occurrence network. In addition, the top 10 co-occurrence keywords include “sleep,” “intensive-care-unit,” “mechanically ventilated patient,” “critically-ill patient,” “light,” “postoperative delirium,” “sedation,” “ICU,” and “melatonin” (see Figure 8B).

To gain a deeper understanding of the research hotspots and topics within this field, we plotted the top 25 keywords with the highest citation burst rate (as shown in Figure 8C) and thematic maps (as illustrated in Supplementary Figure 8D). In Figure 8C, the keyword “dementia” exhibited the longest sustained citation burst, attracting widespread attention during the period from 1994 to 2015. It is of particular note that the keyword “randomized controlled trial” with the highest burst intensity emerged in 2014, to some extent reflecting the growing emphasis placed by researchers on scientifically rigorous experimental design and contributing to the progress of research on light-based delirium treatment interventions. In recent years, “intervention,” “light” and “interventions” have emerged, with their duration continuing through 2024, as well as “pain,” “risk factors,” “validity,” “intensive care units,” “eye mask” and “impact.” It shows that these topics are becoming the new focus of research. In the thematic map (Supplementary Figure 8D), the horizontal axis denotes centrality, that is, the degree of relevance of the topic to the field; the vertical axis denotes density, which represents the extent to which the topic has advanced within the field. Accordingly, four quadrants are divided. Motor Themes in the first quadrant represent both important and well-developed themes, such as older-adults, children, and cognitive dysfunction. The second quadrant has Niche Themes that are better developed but less important to the current field, such as septic shock, cortex, etc. The third quadrant (Emerging or Declining Themes) consists of less developed, marginal themes that may either not be well-established or are just emerging or on the verge of disappearing, such as general anesthesia and memory. The fourth quadrant’s Basic Themes are those that are important to the field but have not been well developed, such as dementia, delirium, sleep, melatonin, etc.

## 4 Discussion

### 4.1 General information

In this study, we performed an in-depth search of the core database of the Web of Science and compiled a collection of 220 academic articles pertaining to the application of light in the treatment of delirium. We observed that, based on key shifts in publication trends and incorporating key advances in the field, 2008 was an inflection point for annual publication growth, and 2017 was a key period for further growth (Figure 2). After 2008, the number of publications exhibited a gradual increase, which may be related to the publication of relevant clinical guidelines during this period. For instance, the 2013 clinical practice guideline on the management of delirium (15), which included phototherapy as an adjunctive intervention, spurred an increase in the number of small-scale studies published. After 2017, with the development of new lighting systems and several high-quality clinical trials driving a large number of research papers, resulting in a significant increase in article production in the third stage. Overall, the number of annual publications in the field shows a steady upward trend.

Through an analysis of the number of publications and total citation counts, we can objectively evaluate the authors who have made the most significant contributions and exerted the greatest influence in the field. Shehabi Yahya from the University of Malaysia and Knauert Melissa P from Yale University in the United States, ranked first in terms of the number of published papers. Among them, Shehabi Yahya not only published the greatest number of papers but also had the highest citation count and H-index, demonstrating his substantial academic influence in the field. Although Egerod Ingrid did not have the highest number of publications, their H-index reached 4, indicating the high quality of their published works and making them worthy of in-depth study by colleagues. However, the co-authorship network analysis (Figure 3B) uncovers the insufficient collaboration and communication among scholars, with most cooperation within the field being limited to internal interactions. This is also evident in the analysis of cooperation networks between countries and institutions (Figures 5C, 6B), where, although there is cooperation between countries and institutions, it is restricted in both scope and depth. In terms of the number of publications, citation frequency, and major institutional cooperation and exchanges, European and American countries, led by the United States, hold a dominant position (Table 3; Figure 5C). This highlights their leading role in the research of this field and their substantial academic accumulation, positioning them as the main driving force behind the field’s development. Therefore, given the disparities in research scope depth among different countries, institutions, and authors, as well as the limited cooperation and exchange, institutions and scholars worldwide should allocate more resources to relevant research. They can strengthen international cooperation by organizing scholar exchange visits between universities, facilitating online learning exchanges, establishing transnational research groups, or conducting hospital field visits, thereby overcoming geographical and political barriers. Additionally, continuous efforts should be made to explore and enhance academic exchanges and cooperation with international peers, aiming to expand the scope and deepen the understanding of research in this field.

Regarding journals related to this field, acute critical care journals have demonstrated strong growth potential, which may be related to the clinical setting in which delirium mainly occurs in the intensive care unit and the critically ill patient population. Notably, the majority of the top 10 most productive journals and the top 10 most-cited journals had an impact factor (IF) < 5.000, which means that publishing research on illumination in delirium in higher-quality journals becomes a challenge.

### 4.2 Research structure, current status, and future trends

#### 4.2.1 Relationship between delirium and light

Delirium is a prevalent clinical syndrome among critically ill and postoperative patients, and its occurrence may be associated with multiple factors, such as the patient’s pre-existing disease, environmental factors, and the medications administered during surgery or anesthesia (7). Particularly in the intensive care unit (ICU), the incidence of delirium is higher, which may be related to specific environmental factors. These factors include the patient’s fear of mortality, potential long-term loss of function, prolonged mechanical ventilation, administration of sedative medications, absence of family support, sleep disturbances, and noise disturbances (16). Therefore, by intervening and improving these risk factors, it is possible to decrease the incidence of delirium, of which sleep deprivation is a potentially modifiable factor (17).

Although there is no direct causal relationship between sleep deprivation and delirium, research indicates that approximately 75% of individuals with delirium experience disrupted sleep patterns (18). Particularly in critically ill patients, alterations in sleep patterns can result in abnormal melatonin levels and disruption of circadian rhythms (19). The regulatory center of circadian rhythms is situated in the suprachiasmatic nucleus (SCN) within the anterior region of the hypothalamus. Approximately 20,000 neurons in this area receive signals from the environment (9). When blue light (wavelength 460–480 nm) irradiates the photoreceptors of the retina, the SCN inhibits the secretion of melatonin by the pineal gland. This blue light predominantly appears in the morning. In the absence of blue light, the secretion of melatonin is enhanced, which promotes sleep (20). Therefore, appropriate light can reinforce and regulate the circadian rhythms, enhance wakefulness and alertness during the day, and facilitate sleep at night (21). It also aids in synchronizing the endogenous circadian rhythm with the 24-h environment cycle, thereby regulating the sleep and awakening of the organism (22–24).

Light therapy is therefore seen as a potential intervention to improve or reduce the incidence of delirium. Building on this understanding, numerous researchers have investigated approaches to decrease the incidence of delirium from the perspectives of light, sleep disturbances, and circadian rhythms. For instance, by studying the relationship between phototherapy and circadian rhythm, Tobias Pustjens et al. (9) discovered that the sole use of dynamic light as a preventive measure has a limited preventive impact on delirium and requires integration with other multi-strategy approaches. A randomized controlled trial conducted by Koen S Simons et al. (25) also demonstrated that high-intensity dynamic light alone did not reduce the cumulative incidence of delirium. Therefore, light therapy should be evaluated as part of a multi-component strategy, such as a combination of ear plugs to reduce noise, adjust sedation strategies, improve the environment, and improve sleep quality, which is consistent with high-frequency keywords derived from keyword co-occurrence network analysis.

#### 4.2.2 The potential for the use of light in delirium

There is no doubt that light therapy is an avenue worth exploring for nonpharmacologic interventions for delirium. Among the top 25 keywords with citation bursts (Figure 8C), the citation burst of the keyword “light” continues until 2024, indicating that the topic of “light” is still being explored and researched.

A systematic review and meta-analysis of randomized clinical trials (26) found that strategies focusing on sleep and circadian health may prevent postoperative delirium, and in particular, timed bright light exposure (*p* = 0.006) showed potential to reduce postoperative delirium. Furthermore, a randomized controlled trial carried out by Chenjun Zou et al. (27) also demonstrated that light therapy had a positive effect on delirium in elderly patients with Alzheimer’s disease-related dementia. The Confusion Assessment Method (CAM) scores decreased at weeks 2 and 4 following light therapy, and ultimately, a 4-week period of light therapy was effective in suppressing delirium in patients with Alzheimer’s disease. In a multi-component improvement study in the ICU ward (28), a novel dynamic lighting system was developed for individualized lighting therapy for patients, in addition to measures such as noise reduction, cognitive training, and workflow optimization. The final results indicate that these improvements may help reduce the incidence and severity of delirium and that integrating a sufficiently intense dynamic lighting system (DLS) into the ward may influence delirium outcomes in critically ill patients by modulating circadian rhythms and melatonin levels.

In conclusion, light-based therapies have demonstrated positive effects in both influencing delirium and interventional treatments, and they possess significant potential for clinical application and in-depth exploratory research.

#### 4.2.3 Future development trends

Based on the analysis presented in Figure 8C and Supplementary Figure 8D, it can be anticipated that future research in the field of delirium will predominantly concentrate on specific research subjects, multi-factor interventions, and fundamental scientific investigations.

To begin with, the subjects will pay special attention to the elderly, children/infants, and hospitalized patients. With the progressive aging of the global population (29, 30), elderly patients, who are a high-risk group for delirium (31), will draw more research interest. For instance, Yan-Yan Wang (32) and Huili Shen et al. (33) carried out relevant studies on living plans for hospitalized elderly patients. These studies involved implementing measures like phototherapy and enhancing sleep quality to investigate their impact on the occurrence of delirium. The studies ultimately concluded that living plans for the elderly can help reduce postoperative delirium, preserve physical and cognitive functions, and decrease hospital length of stay. However, according to the results of the article retrieval and analysis, there are relatively few in-depth studies on pediatric delirium, and most of the subjects of light therapy interventions are adult patients with delirium, which indicates that there is great research potential in the field of childhood delirium. In addition, although research on delirium mostly focuses on severe and postoperative patients, there are relatively few studies on light therapy in hospitalized patients, which is also an area worthy of further exploration.

Second, owing to the current absence of specific treatments for delirium, identifying and targeting risk factors for delirium becomes an effective way to manage delirium. The risk factors for delirium are numerous and complex, including age, underlying medical conditions, medication use, and infections (7). Therefore, both pharmacological and non-pharmacological interventions, such as phototherapy, the use of eye masks and earplugs, environmental adjustments, and psychological and social support, are necessary, taking into account an individual’s multiple risk factors. With the development of machine learning technology and predictive model algorithms, the construction of predictive models to achieve real-time monitoring and advance prediction can be more effective in multi-factor intervention and improve the effectiveness of delirium management. Sandeep R. Pagali et al. (34) used LASSO penalty logistic regression analysis to extract delirium risk factors from a large cohort of heterogeneous patients and developed an MDP model. Chie Nagata and others (35) have also used machine learning algorithms to develop delirium risk prediction models, which play a crucial role in the early identification of high-risk patients and the implementation of preventive strategies. Therefore, the use of models to identify patients’ risk of delirium in advance, and targeted intervention, may become a future research hotspot.

Finally, based on the analysis of the cited articles, there are relatively few fundamental studies investigating the relationship between light exposure and delirium. It might be associated with the intricate pathophysiological mechanism of delirium, which involves neurotransmitters, neuroinflammation, neuronal aging, and endothelial cell dysfunction (36). Some have tested the functional role of the circadian rhythm protein Phase 2 (PER2) in different mouse models similar to delirium (37). Some have discussed the functional organization, and major input and output signals of the circadian system (38). It is undeniable that fundamental research is of great significance for a comprehensive understanding of the pathogenesis of delirium and the fundamental relationship between delirium and light. However, the essential relationship between delirium and light, as well as the interaction mechanism, still needs to be elucidated. Therefore, there is still a lot of room to explore the fundamental research between light and delirium.

## 5 Limitation

Firstly, we statistically analyzed the past research trends, hot topics, and development trends of light applied to delirium through three software programs, namely CiteSpace, VOSviewer, and R-bibliometrix (39), and there are still many limitations in the discussion section.

Secondly, taking into account the stringent journal inclusion criteria and disciplinary authority of Web of Science (WoS), we selected the core collection of WoS to guarantee data quality. However, this single-database approach might result in the omission of relevant research indexed on non-WOS platforms (such as Scopus) or non-SCI journals. Future research could incorporate complementary databases (such as Scopus) to validate the robustness of the conclusions.

Finally, this paper focuses solely on English language literature, and non-English language literature was excluded from the inclusion criteria.

## 6 Conclusion

Through a bibliometric analysis of the literature within the core database of the Web of Science, this paper comprehensively and systematically reviews the application of light in the treatment of delirium. We have observed that the research in this field is expanding rapidly, attracting the involvement of researchers from a greater number of countries and regions globally, and simultaneously garnering more attention from academic journals. By conducting an in-depth exploration of keywords, we discovered that light therapy targeting specific populations like the elderly and children, multi-factor interventions, and fundamental scientific research could represent future research trends, and these areas possess significant development potential.

Overall, while the research in this field continues to advance, greater efforts are required to establish a more comprehensive research framework, which can provide a robust scientific foundation for the application of light therapy in treating delirium.

## Data Availability

The original contributions presented in the study are included in the article/Supplementary material, further inquiries can be directed to the corresponding authors.
