# Supplementary figures and images for: Application of light in delirium: a bibliometric analysis
Source: Front Neurol. 2025 Jul 2;16:1549396. doi: 10.3389/fneur.2025.1549396 (PMC12263596; doi:10.3389/fneur.2025.1549396)

# Supplementary Material

## Supplementary Figures

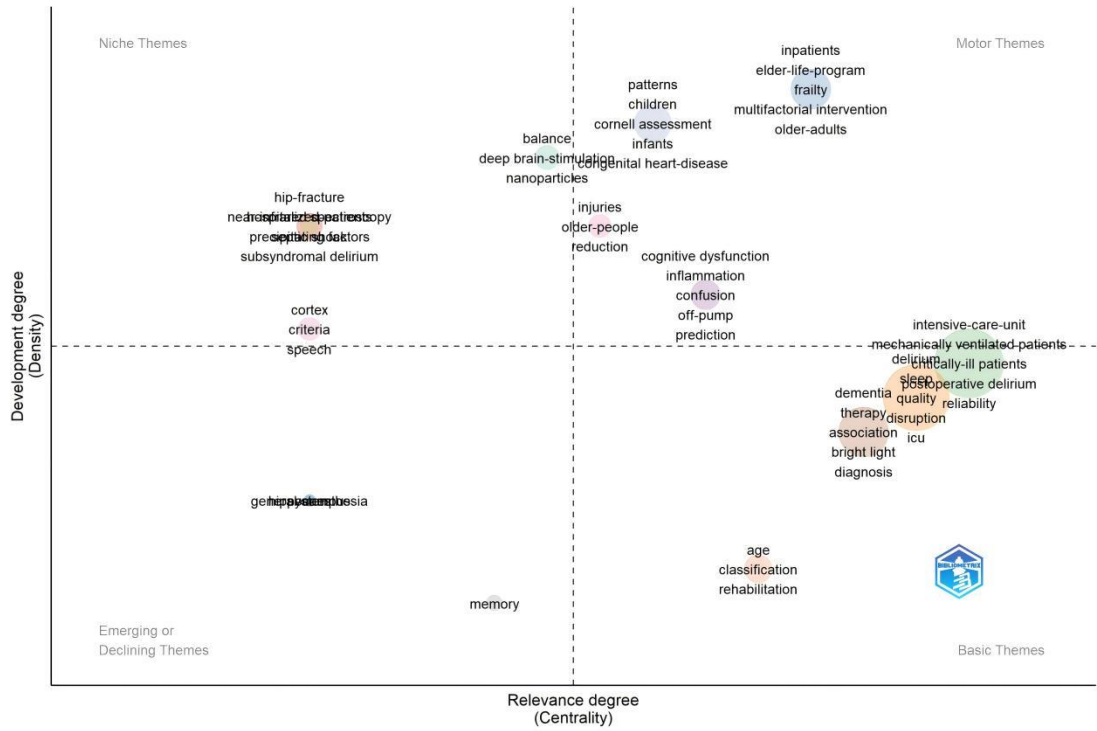

Supplementary Figure S1 (Figure.8D) Thematic map.

Supplement: Supplementary file 1 [file Data_Sheet_1.pdf]
